# Blood-based NfL: A biomarker for differential diagnosis of parkinsonian disorder

**DOI:** 10.1212/WNL.0000000000003680

**Published:** 2017-03-07

**Authors:** Oskar Hansson, Shorena Janelidze, Sara Hall, Nadia Magdalinou, Andrew J. Lees, Ulf Andreasson, Niklas Norgren, Jan Linder, Lars Forsgren, Radu Constantinescu, Henrik Zetterberg, Kaj Blennow, For the Swedish BioFINDER study

**Affiliations:** From the Clinical Memory Research Unit (O.H., S.J., S.H.), Department of Clinical Sciences, Lund University; Memory Clinic (O.H., S.J., S.H.), Skåne University Hospital, Sweden; UCL Institute of Neurology (N.M., A.J.L., H.Z.), Queen Square, London, UK; Clinical Neurochemistry Laboratory (R.C., H.Z., K.B.), Institute of Neuroscience and Physiology (U.A.), The Sahlgrenska Academy at University of Gothenburg, Mölndal; UmanDiagnostics (N.N.), Umeå; and Department of Pharmacology and Clinical Neuroscience (J.L., L.F.), Umeå University, Sweden.

## Abstract

**Objective:**

To determine if blood neurofilament light chain (NfL) protein can discriminate between Parkinson disease (PD) and atypical parkinsonian disorders (APD) with equally high diagnostic accuracy as CSF NfL, and can therefore improve the diagnostic workup of parkinsonian disorders.

**Methods:**

The study included 3 independent prospective cohorts: the Lund (n = 278) and London (n = 117) cohorts, comprising healthy controls and patients with PD, progressive supranuclear palsy (PSP), corticobasal syndrome (CBS), and multiple system atrophy (MSA), as well as an early disease cohort (n = 109) of patients with PD, PSP, MSA, or CBS with disease duration ≤3 years. Blood NfL concentration was measured using an ultrasensitive single molecule array (Simoa) method, and the diagnostic accuracy to distinguish PD from APD was investigated.

**Results:**

We found strong correlations between blood and CSF concentrations of NfL (ρ ≥ 0.73–0.84, *p* ≤ 0.001). Blood NfL was increased in patients with MSA, PSP, and CBS (i.e., all APD groups) when compared to patients with PD as well as healthy controls in all cohorts (*p* < 0.001). Furthermore, in the Lund cohort, blood NfL could accurately distinguish PD from APD (area under the curve [AUC] 0.91) with similar results in both the London cohort (AUC 0.85) and the early disease cohort (AUC 0.81).

**Conclusions:**

Quantification of blood NfL concentration can be used to distinguish PD from APD. Blood-based NfL might consequently be included in the diagnostic workup of patients with parkinsonian symptoms in both primary care and specialized clinics.

**Classification of evidence:**

This study provides Class III evidence that blood NfL levels discriminate between PD and APD.

The differential diagnosis between Parkinson disease (PD) and atypical parkinsonian disorders (APD), i.e., multiple system atrophy (MSA), progressive supranuclear palsy (PSP), and corticobasal degeneration (CBD), is often difficult due to the overlapping symptomatology, especially during the early stages of the disease course.^1,2^ Although there are not yet any established diagnostic methods that can reliably separate PD from APD, neurofilament light chain (NfL) protein in CSF is a promising marker for APD. Our group and others have previously shown that the CSF concentration of NfL is increased in APD but not in PD^3,4^ and that NfL in CSF can discriminate between PD and APD with a high degree of diagnostic accuracy.^5–7^ However, lumbar puncture is not easily implemented in the primary care setting and many patients feel apprehensive about lumbar puncture, reducing the clinical usefulness of these findings. We recently developed an ultrasensitive single molecule array (Simoa) immunoassay for NfL, which is by the far the most sensitive platform for detection of NfL, and it provides better agreement between CSF and blood-derived NfL values compared to other analytical platforms.^8^ We therefore investigated if the levels of NfL in blood are increased in study participants with APD (i.e., PSP, MSA, and CBD) compared with patients with PD and healthy controls in 3 different cohorts including one cohort with early-stage disease. We also investigated whether blood-based NfL can discriminate between PD and APD with the same high diagnostic accuracy as CSF NfL.

## METHODS

### Standard protocol approvals, registrations, and patient consents.

The study was conducted in accordance with the Declaration of Helsinki and was approved by the regional ethical review boards in Lund, London, Umeå, and Gothenburg. All participants provided written informed consent to participate in the study.

### Primary research questions/classification of evidence.

Our primary research question was to determine whether blood NfL can discriminate between PD and APD. The study provides Class III evidence that blood NfL levels discriminate between PD and APD with high diagnostic accuracy.

### Participants.

#### Cohort 1 (Lund cohort).

Patients with PD (n = 171), MSA (n = 30), PSP (n = 19), or corticobasal syndrome (CBS, n = 5) and neurologically healthy controls (n = 53) were enrolled as part of the prospective and longitudinal Swedish BioFINDER study (biofinder.se). In this convenience series, study participants were recruited at the Neurology Clinic, Skåne University Hospital, Lund, Sweden, between 2008 and 2014.^9,10^ The diagnostic criteria for cohort 1 are described in the e-Methods at Neurology.org.

#### Cohort 2 (London cohort).

Patients with PD (n = 20), MSA (n = 30), PSP (n = 29), or CBS (n = 12) and neurologically healthy controls (n = 26) were enrolled at clinics at the National Hospital for Neurology and Neurosurgery, Queen Square, London. The study is prospective and longitudinal in nature. Participants were consecutively enrolled over a 2-year period from 2011 to 2013. The diagnostic criteria for cohort 2 are described in the e-Methods at Neurology.org.

In both cohorts, healthy controls underwent a thorough clinical, neurologic, and cognitive examination. Individuals with cognitive impairment or parkinsonian symptoms and signs were not included in the control group.

#### Cohort 3 (early disease cohort).

Cohort 3 included patients with PD (n = 53), MSA (n = 28), PSP (n = 22), or CBS (n = 6) with early-stage disease (disease duration ≤3 years). The recruitment and demographic characteristics of cohort 3 are described in the e-Methods.

### Biochemical analyses.

Blood and CSF sampling procedures are described in the e-Methods. A sensitive sandwich method (NF-light^®^ ELISA kit; UmanDiagnostics AB, Umeå, Sweden) was used to measure CSF NfL as previously described.^11,12^ NfL concentrations in blood were measured using the monoclonal antibodies and calibrator from the NfL assay, transferred onto the Simoa platform using a homebrew kit (Quanterix; Lexington, MA), as previously described.^13^ Details of the assay performance are given in the e-Methods.

### MRI.

In the Lund cohort, a subgroup of 102 study participants, including 39 controls, 89 patients with PD, 7 patients with PSP, 8 patients with MSA, and 2 patients with CBS, underwent MRI (described in the e-Methods).

### Statistical analyses.

SPSS (IBM, Armonk, NY) was used for statistical analysis. One outlier with plasma NfL value above 10 SD of the mean was excluded from the analysis. Associations between 2 continuous variables were tested with Spearman correlation or linear regression models. Group differences were assessed with Mann-Whitney test and univariate general linear analysis. Regression models were used to adjust for the confounding effects of age and sex. The diagnostic accuracy of blood NfL was examined using receiver operating characteristic curve analysis. A detailed description of the statistical analysis is given in the e-Methods. *p* ≤ 0.05 Was considered statistically significant.

## RESULTS

### Demographics.

Demographic and clinical data for the Lund and London cohorts are shown in tables 1 and 2, respectively. In the Lund cohort, blood levels of NfL correlated with age in the whole cohort (ρ = 0.449, *p* < 0.001), in controls (ρ = 0.436, *p* = 0.001), and in patients with PD (ρ = 0.577, *p* < 0.001), but not in patients with APD (i.e., PSP, MSA, or CBS). Blood levels of NfL were higher in women than in men in the whole population (*p* = 0.041) and in patients with PD (*p* = 0.040). We found similar results in the London cohort, where blood levels of NfL correlated with age in the whole cohort (ρ = 0.290, *p* = 0.001), in controls (ρ = 0.411, *p* = 0.037), and in patients with PD (ρ = 0.483, *p* = 0.031), but not in patients with APD. Blood levels of NfL were higher in women than in men in patients with APD only (*p* = 0.048).

**Table 1:**
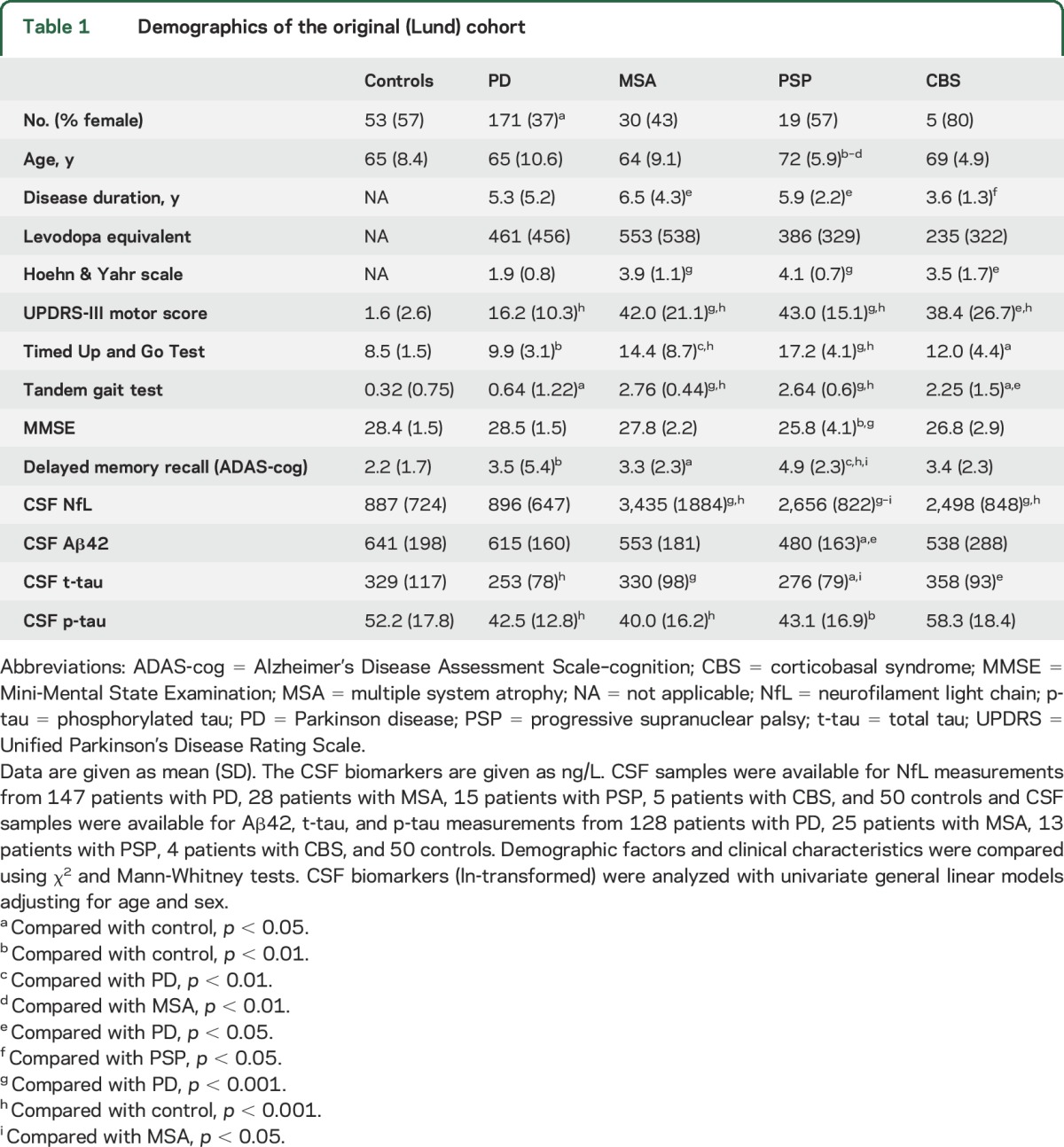
Demographics of the original (Lund) cohort

**Table 2:**
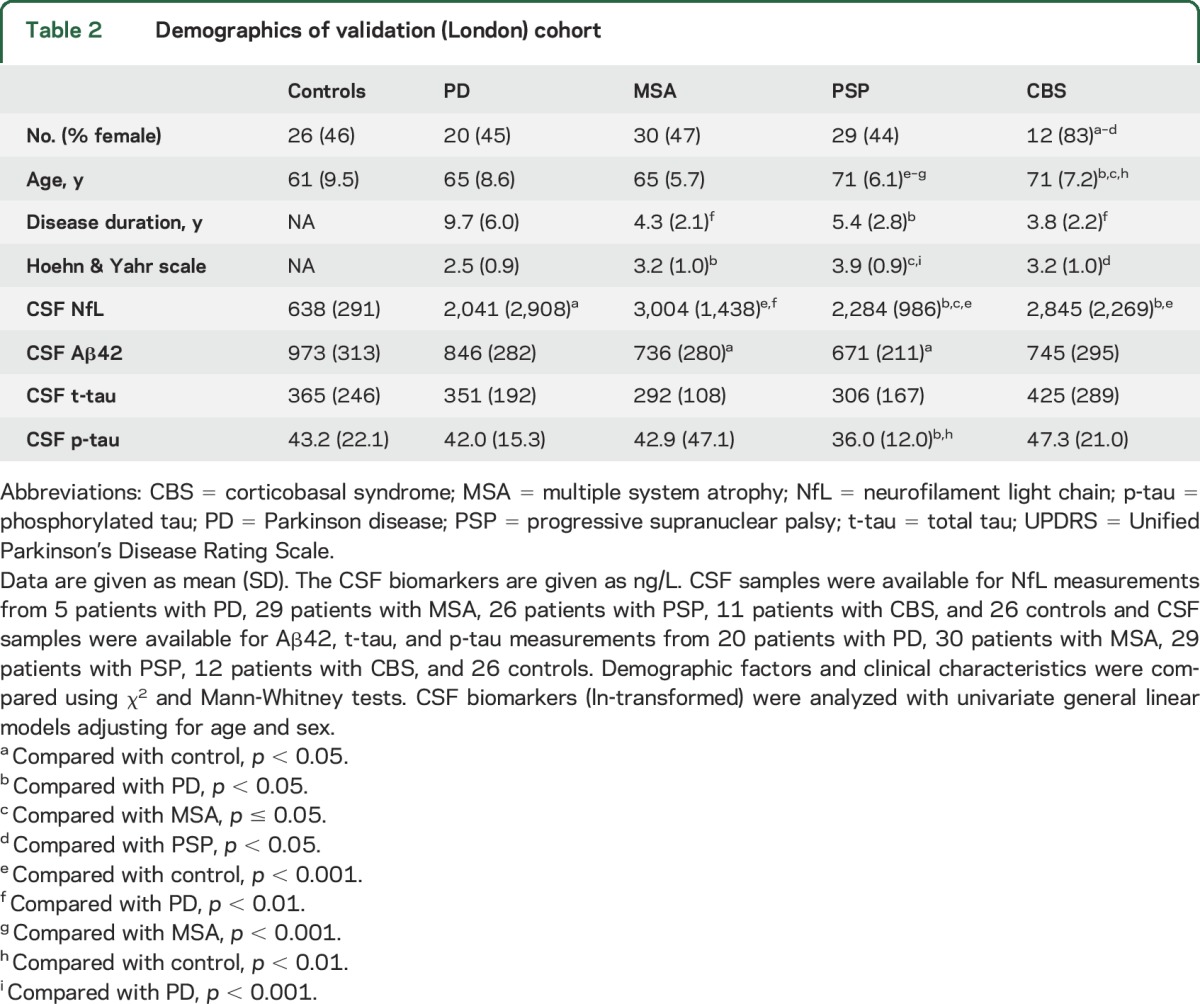
Demographics of validation (London) cohort

Because of the association between blood NfL and age and sex, we additionally adjusted statistical analysis as described below for the confounding effects of these factors.

### Correlations between the levels of NfL in CSF and blood.

#### The Lund cohort.

**Figure 1:**
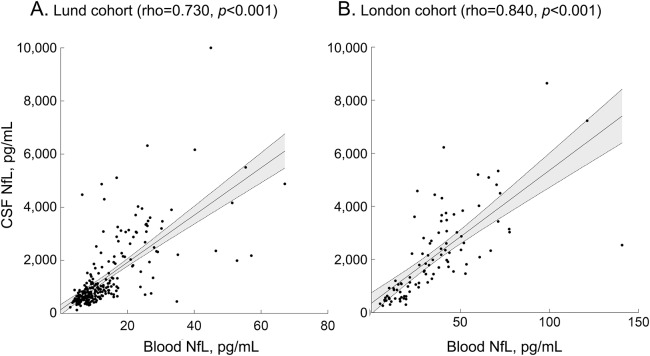
Correlations between blood and CSF levels of neurofilament light chain (NfL) Blood and CSF NfL concentrations were measured in the Lund (A) and London (B) cohorts. NfL measurements of matched CSF samples were available for 245 participants in the Lund cohort (147 Parkinson disease [PD], 28 multiple system atrophy [MSA], 15 progressive supranuclear palsy [PSP], 5 corticobasal syndrome [CBS], and 50 controls) and 97 participants in the London cohort (5 PD, 29 MSA, 26 PSP, 11 CBS, and 26 controls).

Blood NfL correlated strongly with CSF NfL in the whole cohort (ρ = 0.730, *p* < 0.001, figure 1A). The correlations were moderate within individual diagnostic groups most likely due to the smaller sample size: controls, ρ = 0.572, *p* < 0.001; PD, ρ = 0.589, *p* < 0.001; APD, ρ = 0.419, *p* = 0.003. Blood NfL correlated with CSF NfL also when adjusting for the confounding effects of age and sex (the whole cohort, β = 0.697, *p* < 0.001; controls, β = 0.344, *p* = 0.033; PD, β = 0.481, *p* < 0.001; APD, β = 0.523, *p* < 0.001).

#### The London cohort.

Supporting our findings in the Lund cohort, blood NfL correlated strongly with CSF NfL in the total sample (ρ = 0.840, *p* < 0.001, figure 1B). The correlations were moderate within individual diagnostic groups: controls, ρ = 0.406, *p* = 0.040; APD, ρ = 0.599, *p* < 0.001. The number of PD cases with available CSF NfL measurements (n = 5) was too low to conduct statistical analysis. Linear regression analysis revealed associations between blood and CSF levels of NfL also when adjusting for confounding effect of age and sex (the whole cohort, β = 0.796, *p* < 0.001; controls, β = 0.449, *p* = 0.022; APD, β = 0.677, *p* < 0.001).

### Blood levels of NfL in the different diagnostic groups.

#### The Lund cohort.

**Figure 2:**
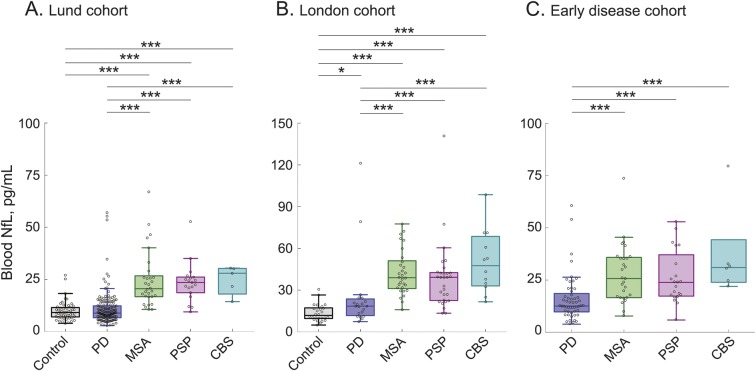
Blood neurofilament light chain (NfL) in different diagnostic groups Blood concentrations of NfL in the Lund (A), London (B), and early disease (C) cohorts; *p* values are from univariate general linear models adjusting for age and sex; *p < 0.05; **p < 0.01; ***p < 0.001. One progressive supranuclear palsy (PSP) case in the early disease cohort with NfL concentration of 134.3 pg/mL is not shown in C (but was included in all statistical analysis). CBS = corticobasal syndrome; MSA = multiple system atrophy; PD = Parkinson disease.

The levels of blood NfL were clearly increased in PSP, MSA, and CBS when compared to controls (PSP, *p* < 0.001; MSA, *p* < 0.001; CBS, *p* < 0.001) and patients with PD (PSP, *p* < 0.001; MSA, *p* < 0.001; CBS, *p* < 0.001), with all analyses adjusted for age and sex (figure 2A). The significant differences in blood NfL between the PD and APD groups were not affected when additionally controlling for disease duration (all *p* < 0.001).

#### The London cohort.

The results obtained in the London cohort were similar to those obtained in the Lund cohort (figure 2B). The levels of blood NfL were clearly increased in PSP, MSA, and CBS when compared to controls (PSP, *p* < 0.001; MSA, *p* < 0.001; CBS, *p* < 0.001) and patients with PD (PSP, *p* = 0.001; MSA, *p* < 0.001; CBS, *p* < 0.001). In addition, we found that in the London cohort, blood NfL was increased in patients with PD compared with controls (*p* = 0.011) but the levels were far below those observed in patients with APD (figure 2B). The significant differences in blood NfL between the PD and APD groups were not affected when additionally controlling for disease duration (PSP, *p* = 0.011; MSA, *p* = 0.002; CBS, *p* < 0.001).

#### The early disease cohort.

Because differential diagnosis of parkinsonian disorders is particularly challenging during the early disease stages, we included a third cohort of patients who all had a disease duration between 0 and 3 years. In agreement with the data in the Lund and London cohorts, the levels of blood NfL were increased in PSP (*p* < 0.001), MSA (*p* < 0.001), and CBS (*p* < 0.001) compared to patients with PD already early in the disease (figure 2C).

### The diagnostic accuracy of blood NfL.

#### The Lund cohort.

**Figure 3:**
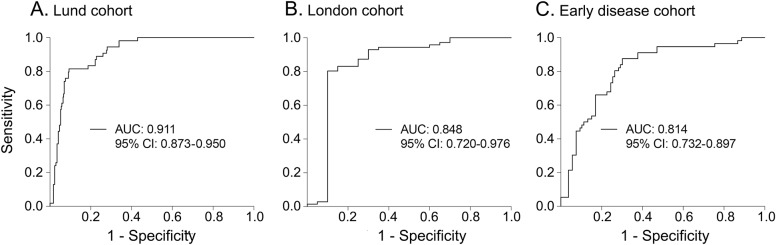
Receiver operating characteristic (ROC) curves of blood neurofilament light chain (NfL) ROC curves of blood NfL to distinguish Parkinson disease (PD) from atypical parkinsonian disorders (APD) (progressive supranuclear palsy [PSP], multiple system atrophy [MSA], and corticobasal degeneration [CBD]) in the Lund (A), London (B) and early disease (C) cohorts. The early disease cohort included 2 patient groups that were recruited in Göteborg and Umeå, respectively (e-Methods). ROC analysis produced similar results when diagnostic accuracy of blood NfL for differentiating PD from APD was assessed in Göteborg (area under the curve [AUC] 0.80) and Umeå (AUC = 0.81) patients separately.

Blood NfL distinguished patients with PD from patients with APD with high accuracy (area under the curve [AUC] 0.91, 95% confidence interval [CI] 0.87–0.95; figure 3A) and with a specificity of 91% and a sensitivity of 82%. The results were similar when individual APD groups (PSP, MSA, and CBS) were analyzed separately (table e-1). Blood NfL could also accurately separate patients with PD and controls grouped together from APD (AUC 0.92, 95% CI 0.88–0.95, specificity 92%, sensitivity 82%). The diagnostic accuracy for blood NfL was as high as for CSF NfL (table e-2).

#### The London cohort.

Likewise, in the London cohort, blood NfL could differentiate PD from APD (AUC 0.85, 95% CI 0.72–0.98; figure 3B) with a specificity of 90% and a sensitivity of 80%. The results were similar when individual APD groups (PSP, MSA, and CBS) were analyzed separately (table e-1). The diagnostic accuracy was even higher when separating APD from PD and controls (AUC 0.91, 95% CI 0.85–0.98, specificity 83%, sensitivity 93%).

#### The early disease cohort.

Similar to the Lund and London cohort, blood NfL accurately distinguished PD from APD in patients with early-stage disease (AUC 0.81, 95% CI 0.73–0.90, 80% specificity, 70% sensitivity; figure 3C). The results were comparable when individual APD groups (PSP, MSA, and CBS) were analyzed separately (table e-1).

### Correlations among blood NfL, CSF biomarkers, and clinical symptoms.

#### The Lund cohort.

First we studied the associations of blood NfL and other established CSF biomarkers than CSF NfL. All analyses below were adjusted for age and sex. In the whole cohort, we found that higher levels of blood NfL were associated with lower levels of CSF Aβ42 (β = −0.201, *p* = 0.001) and higher levels of CSF tau (β = 0.151, *p* = 0.020), but not phosphorylated tau (p-tau). There were no differences in blood NfL levels between participants with pathologic compared to normal CSF Aβ42 levels (controls, *p* = 0.126; PD, *p* = 0.490; APD, *p* = 0.181).

Next we studied the associations between blood NfL and clinical characteristics in the PD and APD cohorts, respectively. In the PD cohort, we found that blood NfL correlated with disease duration (β = 0.278, *p* < 0.001), the levodopa equivalent (β = 0.235, *p* = 0.003), Hoehn & Yahr stage (β = 0.187, *p* = 0.004), Unified Parkinson’s Disease Rating Scale (UPDRS)–III motor score (β = 0.227, *p* < 0.001), Timed Up and Go Test (β = 0.164, *p* = 0.036), and tandem gait test (β = 0.237, *p* = 0.045); i.e., higher blood NfL levels were associated with longer disease duration and more aggravated motor symptoms in PD (blood NfL concentrations across UPDRS-III, Hoehn & Yahr, and disease duration ranges are shown in table e-3). However, in APD, blood NfL only correlated with Hoehn & Yahr stage (β = 0.286, *p* = 0.040) and UPDRS-III motor score (β = 0.449, *p* = 0.001), but not with disease duration or the other clinical assessments.

#### The London cohort.

In the London cohort, higher levels of blood NfL were associated with lower levels of CSF Aβ42 in the whole cohort (β = −0.182, *p* = 0.034), but not with CSF tau and CSF p-tau. In PD and APD groups, blood NfL did not correlate with disease duration or Hoehn & Yahr stage.

#### The early disease cohort.

Blood NfL was not associated with either disease duration or Hoehn & Yahr stage in PD and APD groups (table e-4).

### Correlations between blood NfL and white matter lesions (WMLs).

#### The Lund cohort.

There were no differences in WMLs between the diagnostic groups (Fazekas total score, mean ± SD; controls, 3.8 ± 2.8; PD, 3.9 ± 3.3; and APD, 4.1 ± 3.3). Increased blood NfL was associated with higher Fazekas scores in patients with PD (ρ = 0.328, *p* = 0.002) but not in the control or APD groups (ρ = 0.309, *p* = 0.056 and ρ = 0.341, *p* = 0.181, respectively). However, the correlation in patients with PD appeared to be confounded by the effects of age and sex as linear regression models adjusting for these variables did not show associations between blood NfL and WMLs.

## DISCUSSION

The results of the present study strongly indicate that NfL when measured in blood can be used to distinguish between patients with PD and patients with PSP, MSA, and CBS with high diagnostic accuracy (AUCs 0.81–0.91). The diagnostic accuracy for NfL in blood was as high as that obtained when using CSF NfL. The diagnostic utility of blood NfL to separate patients with PD from patients with APD has not been studied previously, but several publications have shown that the levels of NfL in CSF can be used to distinguish between these disorders with AUCs of about 0.85–0.90.^5–7^ Although a number of other CSF biomarkers and biomarker panels have been suggested to differentiate PD from ADP,^5–7^ blood biomarkers offering sufficient diagnostic accuracy are unavailable besides NfL. The current results confirm that NfL levels in blood, quantified with high-sensitivity immunoassays, reflect the levels in the CSF^13–15^ and that the plasma NfL levels are increased in PSP^16^ and other α-synucleinopathies.^17^

NfL is a marker of degeneration of large myelinated axons.^18^ It is thus increased in CSF of several neurologic disorders, including acute conditions such as stroke^19^ and traumatic brain injury,^20^ as well as in chronic neurodegenerative disorders such as APD (PSP, MSA, and CBD),^3–7^ amyotrophic lateral sclerosis,^14,15,21^ and frontotemporal dementia.^22,23^ CSF and blood NfL are not increased in PD, which could be due in part to less severe and widespread axonal degeneration in PD compared to APD. Diffusion MRI studies have shown extensive white matter damage in PSP, MSA, and CBD, but not in PD.^24–26^ In general, there were higher blood NfL levels in more advanced PD (table e-3); nonetheless, the changes were much more modest compared to those observed in APD. Previous studies on NfL in CSF in patients with APD from our groups show that NfL correlates with disease severity but not disease duration.^6,7^ The lack of association between blood NfL and disease duration indicates that the rate of degeneration of myelinated axons is rather constant throughout the disease course in APD. In this study, we could replicate these findings using the blood test for NfL, further adding to the reliability of blood NfL in APD. These findings together also suggest that NfL in blood may be useful in clinical trials to detect treatment effects on axonal degeneration.

It is often difficult to differentiate clinically between PD and APD, particularly during the early disease stages. For the individual patient and caregiver, a correct and early diagnosis is highly important. Also, with the hope for advanced disease-modifying therapies, early and correct diagnosis is vital to identify the patients who would benefit from certain disease-specific treatments. In several countries, NfL in CSF has over the last decade been shown to be a useful addition to the clinical workup of patients with parkinsonism. However, the clinical usefulness has been limited by the invasiveness of lumbar puncture. Therefore, the finding that NfL in blood correlates with and is equal to NfL in CSF in distinguishing PD from APD may prove to be highly useful in the clinical setting and could even be used in the primary care setting. Further, blood-derived NfL might be used in clinical trials where repeated measures may be necessary to follow the rate of axonal degeneration over time.

Although NfL in CSF as well as in blood can differentiate APD from PD, NfL cannot be used to separate PSP, MSA, and CBD from each other. However, increased levels of blood NfL in a patient with parkinsonism may indicate that the patient has APD and the doctor should look carefully for symptoms and clinical signs supporting PSP, MSA, or CBS. Certain other diagnostic methods, such as MRI or fluorodeoxyglucose PET, might also help to distinguish between these atypical parkinsonian disorders.^27^

The patients with APD included in the Lund and London cohorts had disease durations of 4–6 years on average. From a clinical point of view, it is most important to establish methods that can separate APD from PD during the earliest stages of the disease course. Importantly, the data obtained in the early disease cohort (with symptom onset within 0–3 years) show that blood NfL is increased in APD and can accurately differentiate APD from PD even in patients with a short disease duration.

There are limitations with the present study. The diagnoses of the study participants were based on clinical criteria and not neuropathologic examination. However, the clinical diagnoses were made by medical doctors specialized in movement disorders and the patients were followed over time with reassessments at each follow-up visit. This should increase the diagnostic accuracy, as compared to what can be achieved with diagnoses made at a single visit. In the London cohort, 9 patients eventually underwent neuropathologic examination and the neuropathologic diagnoses confirmed the clinical diagnoses in all 9 cases. Importantly, any potential clinical misdiagnosis in the present study is more likely to result in underestimation, rather than overestimation, of the diagnostic accuracy of blood NfL when separating PD from APD.

Blood biomarkers have previously been suggested to improve the diagnostic accuracy of neurodegenerative disorder like Alzheimer disease or MSA, but subsequent studies have not been able to reproduce the findings.^4,28,29^ This highlights the importance of always using at least 2 independent cohorts obtained at different clinics when evaluating biomarkers. Importantly, in the present study we obtained similar results for blood NfL in 3 independent cohorts, indicating that this is indeed a robust marker for differentiating PD from APD. Absolute concentrations of NfL differed between the cohorts most likely due to lot-to-lot variation in assay performance or matrix effects (plasma vs serum). Thus development of a fully automated clinical grade assay and establishment of cutoff points would be necessary for implementation of blood-based NfL measurements in clinical practice.

The present study has shown in 3 independent cohorts that NfL concentrations in blood are increased in PSP, MSA, and CBS when compared to PD and healthy controls. This easily accessible biomarker of axonal degeneration may improve the diagnostic workup of patients with parkinsonian symptoms in specialized clinics as well as in primary care settings.

## Supplementary Material

Data Supplement

Coinvestigators

Accompanying Editorial

## GLOSSARY

APD: atypical parkinsonian disorders
AUC: area under the curve
CBD: corticobasal degeneration
CBS: corticobasal syndrome
CI: confidence interval
MSA: multiple system atrophy
NfL: neurofilament light chain
p-tau: phosphorylated tau
PD: Parkinson disease
PSP: progressive supranuclear palsy
UPDRS: Unified Parkinson's Disease Rating Scale
WML: white matter lesion
