# Supplementary material for: Blood-based NfL: A biomarker for differential diagnosis of parkinsonian disorder
Source: Neurology. 2017 Mar 7;88(10):930–7. doi: 10.1212/WNL.0000000000003680 (PMC5333515; doi:10.1212/WNL.0000000000003680)
Supplement: Data Supplement [file supp_WNL.0000000000003680_Hansson_et_al_Supplement2_notrackcx.docx]

**Supplemental Data**

**Blood-based NfL: A Biomarker for Differential Diagnosis of Parkinsonian Disorders**

Oskar Hansson, MD, PhD; Shorena Janelidze, PhD; Sara Hall, MD; Nadia Magdalinou, MD; Andrew J Lees, MD, PhD; Ulf Andreasson, PhD; Niklas Norgren, Jan Linder, MD, PhD; Lars Forsgren, MD, PhD; Radu Constantinescu, MD, PhD; PhD; Henrik Zetterberg, MD, PhD; Kaj Blennow, MD, PhD for the Swedish BioFINDER study

**e-Methods**

***Participants***

**Cohort 1 (Lund cohort)**

A medical doctor specialized in movement disorders examined all study participants. A thorough medical history was taken and the patients underwent extensive testing, both regarding motor symptoms and cognition, including e.g. the Unified Parkinson’s Disease Rating Scale (UPDRS),^1^ the Hoehn & Yahr scale,^2^ the Schwab & England (SE) activities of daily living dependency scale,^1^ the Timed Up and Go test,^3^ tandem gait test,^4^ Mini Mental State Examination (MMSE),^5^ the delayed verbal memory part of Alzheimer’s Disease Assessment Scale (ADAS).^6^ The patients with PD met the National Institute of Neurological Disorders and Stroke Diagnostic Criteria for PD.^7^ Patients with MSA met the consensus statement on the diagnosis of that disorder.^8^ Patients with PSP met the criteria according to the report of the National Institute of Neurological Disorders and Stroke–Society for Progressive Supranuclear Palsy International Workshop.^9^ Patients with CBS were diagnosed in accordance with guidelines.^10^

**Cohort 2 (London cohort)**

The patients were investigated by a medical doctor specialized in movement disorders. The same diagnostic criteria were used as for the Lund cohort, except for PD where the Queen Square Brain Bank Criteria was used.^11^

**Cohort 3 (Early disease cohort)**

Cohort 3 included a total of 53 PD, 28 MSA, 22 PSP and 6 CBS patients with early stage disease (disease duration ≤3 years). Patients with PD (n=11), MSA (n=15), PSP (n=11), CBS (n=5) were consecutively enrolled between 2009 and 2013 as part of their clinical follow-up at the Neurological Department, Sahlgrenska University Hospital, Göteborg, Sweden. The patients were investigated by neurologists specialized in movement disorders. The same diagnostic criteria were used as for the London cohort, except for CBD where Lang’s criteria were used.^12^ The rest of the patients (42 PD 13 MSA, 11 PSP and 1 CBS cases) were enrolled either as part of the prospective and longitudinal Swedish New Parkinsonism in Umeå (NYPUM) study, except for nine patients (5 MSA, 3 PSP, 1 CBS) who were enrolled after inclusion into the NYPUM study had ended in 2009 (starting 2004). A medical doctor specialized in movement disorders examined all study participants.^13^ The same diagnostic criteria were used as for the London cohort. Demographic and clinical data for cohort 3 are shown in table e-4.

***Blood and CSF Samples***

Plasma (Lund), serum (London) and CSF (Lund and London) samples were collected with the patients non-fasting. Blood and CSF were drawn on the same day and at the same time of day (between 9:00 and 13:00, blood was obtained within 15 min of CSF sampling). All samples were centrifuged within 30 minutes at -4°C at 2000g for 10 minutes to remove cells and debris. Samples were stored in aliquots at −80°C pending biochemical analysis. The procedure and analysis of the CSF followed the Alzheimer’s Association Flow Chart for CSF biomarkers.^14^ In the "early disease cohort" plasma (Umeå) and serum (Göteborg) were also collected with the patients non-fasting, centrifuged and stored at −80°C after collection.

***Biochemical Analyses***

A sensitive sandwich method (NF-light® ELISA kit, UmanDiagnostics AB, Umeå, Sweden) was used to measure NfL concentrations in CSF as previously described.^15, 16^ The coefficient of variation (CV) was <6% for the repeatability and <7% for intermediate precision. The lower limit of quantification (LLOQ) was 50 pg/mL as defined by repeated measurements (n = 15) of a CSF sample with low concentration (52 pg/mL) and variability (CV = 12%). NfL concentrations in blood were measured using the monoclonal antibodies and calibrator from the NF-light assay, transferred onto the Simoa platform using a homebrew kit (Quanterix, Lexington, MA, USA), as previously described.^17^ The limit of detection (mean blank signal + 3 SD) and the lower limit of quantification (mean blank signal + 10 SD) for the Simoa NfL assay were 0.3 pg/mL and 2.7 pg/mL, respectively. Samples were analyzed in duplicates with CV below 10%. The "early disease cohort" was analyzed separately from the Lund and London cohorts, therefore we included several samples from the Lund cohort to normalize the NfL values.

In cohorts 1 and 2, the CSF levels of Aβ42, and tau phosphorylated at Thr181 (P-tau) were analyzed using INNOTEST ELISA (Fujirebio Europe, Ghent, Belgium). CSF tau was analyzed with the INNOTEST ELISA in the London cohort and the EUROIMMUN ELISA (EUROIMMUN AG, Lübeck, Germany) in the Lund cohort. All analyses were performed by technicians who were blinded to the clinical data.

***Magnetic Resonance Imaging***

In the Lund cohort a subgroup of 102 study participants, including 39 controls, 89 PD, 7 PSP, 8 MSA and 2 CBS patients, underwent MRI using a 3 T Siemens® system (Skyra) equipped with a 20 channel head coil. For assessment of WMLs standard clinical T2 FLAIR (23 slices, slice thickness 5 mm) images were used. Visual rating of WML on FLAIR images according to the Fazekas scale as previously described.^18^

***Statistical Analyses***

SPSS (IBM, Armonk, NY, US) was used for statistical analysis. In Lund cohort, one outlier with plasma NfL value above 10 SD of the mean of the cohort was excluded from the analysis. Correlations between blood and CSF NfL and between blood NfL and age were examined using Spearman's correlation analysis. Gender differences in NfL levels within each diagnostic group were assessed with Mann-Whitney test. We also studied if blood levels of NfL were associated with CSF levels of NfL, Aβ42, Aβ40, tau and p-tau as well as with clinical characteristics of the patients (disease duration, the levodopa equivalent, Hoehn & Yahr stage, UPDRS-III motor score, timed up and go test and tandem gait test) using linear regression models adjusting for age and gender. Regression analyses were conducted for the whole cohorts and for control, PD and APD groups. However, in the London cohort, CSF NfL measurements were only available for 5 PD cases and therefore associations between blood and CSF NfL were not analyzed in these patients. Ln-transformed variables were used for regression analysis. Comparisons of ln-transformed blood levels of NfL between different diagnostic groups were investigated with univariate general linear models adjusting for age and gender. The diagnostic accuracy of blood NfL was assessed using receiving operating characteristic (ROC) curve analysis. To define optimal sensitivity and specificity Youden’s index was calculated for each of the points of the ROC curve. Cases with missing data were excluded from the statistical analysis, p ≤ 0 .05 was considered statistically significant.

**e-References**

1. Fahn S, Elton R, Members of the UPDRS Development Committee. Unified Parkinson’s Disease Rating Scale. In: Fahn S, Marsden CD, Calne DB, Goldstein M, eds. Recent Developments in Parkinson's Disease. Florham Park: NJ. Macmillan Health Care Information, 1987: 153-163.

2. Hoehn MM, Yahr MD. Parkinsonism: onset, progression and mortality. Neurology 1967;17:427-442.

3. Morris S, Morris ME, Iansek R. Reliability of measurements obtained with the Timed "Up & Go" test in people with Parkinson disease. Phys Ther 2001;81:810-818.

4. Abdo WF, Borm GF, Munneke M, Verbeek MM, Esselink RA, Bloem BR. Ten steps to identify atypical parkinsonism. J Neurol Neurosurg Psychiatry 2006;77:1367-1369.

5. Folstein MF, Folstein SE, McHugh PR. "Mini-mental state". A practical method for grading the cognitive state of patients for the clinician. J Psychiatr Res 1975;12:189-198.

6. Mohs RC, Cohen L. Alzheimer's Disease Assessment Scale (ADAS). Psychopharmacol Bull 1988;24:627-628.

7. Gelb DJ, Oliver E, Gilman S. Diagnostic criteria for Parkinson disease. Arch Neurol 1999;56:33-39.

8. Gilman S, Low PA, Quinn N, et al. Consensus statement on the diagnosis of multiple system atrophy. J Neurol Sci 1999;163:94-98.

9. Litvan I, Agid Y, Calne D, et al. Clinical research criteria for the diagnosis of progressive supranuclear palsy (Steele-Richardson-Olszewski syndrome): report of the NINDS-SPSP international workshop. Neurology 1996;47:1-9.

10. Litvan I, Hauw JJ, Bartko JJ, et al. Validity and reliability of the preliminary NINDS neuropathologic criteria for progressive supranuclear palsy and related disorders. J Neuropathol Exp Neurol 1996;55:97-105.

11. Hughes AJ, Daniel SE, Kilford L, Lees AJ. Accuracy of clinical diagnosis of idiopathic Parkinson's disease: a clinico-pathological study of 100 cases. J Neurol Neurosurg Psychiatry 1992;55:181-184.

12. Lang AE, Riley DE, Bergeron C. Cortical-basal ganglionic degeneration. In: Calne DB, ed. Neurodegenerative Diseases. Philadelphia: W.B. Saunders, 1994: 877–894.

13. Backstrom DC, Eriksson Domellof M, Linder J, et al. Cerebrospinal Fluid Patterns and the Risk of Future Dementia in Early, Incident Parkinson Disease. JAMA Neurol 2015;72:1175-1182.

14. Blennow K, Hampel H, Weiner M, Zetterberg H. Cerebrospinal fluid and plasma biomarkers in Alzheimer disease. Nat Rev Neurol 2010;6:131-144.

15. Jessen Krut J, Mellberg T, Price RW, et al. Biomarker evidence of axonal injury in neuroasymptomatic HIV-1 patients. PLoS One 2014;9:e88591.

16. Norgren N, Rosengren L, Stigbrand T. Elevated neurofilament levels in neurological diseases. Brain Res 2003;987:25-31.

17. Gisslén M, Richard WP, Andreasson U, et al. Plasma Concentration of the Neurofilament Light Protein (NFL) is a Biomarker of CNS Injury in HIV Infection: A Cross-Sectional Study. EBioMedicine 2016;3:135-140.

18. Fazekas F, Chawluk JB, Alavi A, Hurtig HI, Zimmerman RA. MR signal abnormalities at 1.5 T in Alzheimer's dementia and normal aging. AJR Am J Roentgenol 1987;149:351-356.

**Table e-1.** Receiver Operating Characteristic (ROC) analysis of blood NfL for distinguishing PD patients from PSP, MSA or CBS patients.

|  | AUC | Sensitivity | Specificity |
| --- | --- | --- | --- |
| ***The Lund Cohort*** |  |  |  |
| PSP | 0.92, 95% CI =0.86-0.97 | 0.84 | 0.91 |
| MSA | 0.91, 95% CI =0.86-0.95 | 0.80 | 0.91 |
| CBS | 0.94, 95% CI =0.88-1.00 | 1.00 | 0.80 |
| ***The London Cohort*** |  |  |  |
| PSP | 0.81, 95% CI =0.67-0.95 | 0.72 | 0.90 |
| MSA | 0.87, 95% CI =0.73-1.00 | 0.97 | 0.90 |
| CBS | 0.88, 95% CI =0.75-1.00 | 0.92 | 0.85 |
| ***The Early Disease Cohort*** |  |  |  |
| PSP | 0.80, 95% CI =0.69-0.91 | 0.86 | 0.72 |
| MSA | 0.81, 95% CI =0.70-0.91 | 0.86 | 0.70 |
| CBS | 0.91, 95% CI =0.83-0.99 | 1.00 | 0.83 |

AUC, area under the curve; CI, confidence interval.

**Table e-2.** Receiver Operating Characteristic (ROC) analysis of CSF NfL for distinguishing PD patients or PD patients and controls grouped together from PSP, MSA or CBS patients in the Lund Cohort.

|  | AUC | Sensitivity | Specificity |
| --- | --- | --- | --- |
| PD and controls  vs APD | 0.95, 95% CI =0.92-0.98 | 0.92 | 0.92 |
| PD vs |  |  |  |
| APD | 0.96, 95% CI =0.92-0.99 | 0.92 | 0.93 |
| PSP | 0.97, 95% CI =0.94-1.00 | 0.93 | 0.95 |
| MSA | 0.95, 95% CI =0.90-0.99 | 0.89 | 0.93 |
| CBS | 0.96, 95% CI =0.93-1.00 | 1.00 | 0.93 |

AUC, area under the curve; CI, confidence interval.

**Table e-3.** NfL levels in PD patients in the Lund and London cohorts.

|  | | Plasma NfL  (the Lund cohort) | Serum NfL  (the London cohort) |
| --- | --- | --- | --- |
| UPDRS-III  0-14  >14 | | 9.2 (4.7), n=87  12.5 (9.7), n=84 | N/A  N/A |
| Hoehn & Yahr  0-2  >2 | | 9.5 (5.0), n=118  11.2 (4.5), n=53 | 21.6 (18.8), n=12  30.9 (37.0), n=8 |
| Disease duration  1-5  >5 | | 9.5 (5.0), n=114  13.7 (11.3), n=57 | 24.1 (25.1), n=7  26.0 (29.0), n=13 |
| CSF Aβ42 **^a^**  normal  pathologic | 9.8 (5.2), n=87  13.0 (10.9), n=41 | | 26.9 (29.1), n=17  16.7 (2.8), n=3 |
| WMLs **^b^**  normal  pathologic | 8.2 (3.8), n=59  10.1 (3.7), n=30 | | N/A  N/A |
| MMSE **^c^**  normal  pathologic | 10.4 (6.5), n=132  12.2 (11.3), n=37 | | N/A  N/A |

Data is given as Mean (standard deviation, n).

^a^ PD patients were categorized into groups with normal and pathological CSF signature using the CSF Aβ42 cutoff≤550pg/ml (the cutoff used in clinical routine).

^b^ Fazekas normal 0-4, pathologic >4

^c^ MMSE normal 28-30, pathologic <28.

N/A, not available; MMSE, Mini Mental State Examination; PD, Parkinson's disease; UPDRS, Unified Parkinson’s Disease Rating Scale (assessed in on-stage); WML, white matter lesions.

**Table e-4.** Demographics of the "early disease cohort".

|  | PD | MSA | PSP | CBS |
| --- | --- | --- | --- | --- |
| Number (female%) | 53 (43%) | 28 (54%) | 22 (64%) | 6 (100%) |
| Age | 65 (12.2) | 66 (9.4) | 70 (8.2) | 65 (12.3) |
| Disease duration | 1.3 (0.8) | 1.9 (0.9) | 2.2 (1.0) | 2.0 (0.9) |
| UPDRS-III* | 23.4 (1.7) | 27.6 (4.0) | 29.6 (4.0) | N/A |
| Hoehn & Yahr scale | 2.0 (0.6) | 3.1 (0.9) | 3.2 (1.0) | 2.8 (1.0) |

Data is given as Mean and Standard deviation (SD).

* UPDRS-III was available for 42 PD, 10, MSA and 9 PSP patients.

CBS, corticobasal syndrome; MSA, multiple system atrophy; N/A, not available; NfL, neurofilament light chain; PD, Parkinson's disease; PSP, progressive supranuclear palsy; UPDRS, Unified Parkinson’s Disease Rating Scale.
