# Supplementary material for: Blood-based NfL: A biomarker for differential diagnosis of parkinsonian disorder
Source: Neurology. 2017 Mar 7;88(10):930–7. doi: 10.1212/WNL.0000000000003680 (PMC5333515; doi:10.1212/WNL.0000000000003680)
Supplement: Coinvestigators [file supp_88_10_930_v2_index.html]

Blood-based NfL — Coinvestigators 

# Blood-based NfL

## Coinvestigators

**Neurology® data supplements are not copyedited before publication. Published editorials and translations have been copyedited.  
 © 2017 American Academy of Neurology.  
  
 Files in this Data Supplement:**

- Coinvestigators - Microsoft Word file
