# Supplementary material for: Blood-based NfL: A biomarker for differential diagnosis of parkinsonian disorder
Source: Neurology. 2017 Mar 7;88(10):930–7. doi: 10.1212/WNL.0000000000003680 (PMC5333515; doi:10.1212/WNL.0000000000003680)
Supplement: Coinvestigators [file supp_WNL.0000000000003680_Hansson_et_al_Supplement_conivestigators.docx]

**The BioFINDER Study Group**

Lennart Minthon, MD PhD (Head of the Clinical Memory Research Unit, Lund University), Håkan Toresson, MD (Cell Biology Core, Lund University), Katarina Nägga, MD PhD (Clinical Core, Lund University), Sebastian Palmqvist, MD PhD (Clinical Core, Lund University), Erik Stomrud, MD PhD (Clinical Core, Lund University), Per Johansson, MD PhD (Clinical Core, Lund University), Christer Nilsson, MD PhD (Clinical Core, Lund University), Maria Nilsson, PhD (Clinical Core, Lund University), Niklas Mattsson, MD PhD (Clinical Core, Lund University), Daniel Lindqvist, MD PhD (Clinical Core, Lund University), Susanna Vestberg, PhD (Neuropsychology Core, Lund University), Shorena Janelidze, PhD (CSF Core, Lund University), Danielle van Westen, MD PhD (MRI Core, Lund University), Jimmy Lätt, PhD (MRI Core, Lund University), Peter Mannfolk, PhD (MRI Core, Lund University), Markus Nilsson, PhD (MRI Core, Lund University), Olof Strandberg, PhD, (MRI Core, Lund University), Pia Sundgren, MD PhD (MRI Core, Lund University), Freddy Ståhlberg, MD PhD (MRI Core, Lund University), Olof Lindberg, PhD (MRI Core, Karolinska Institutet), Eric Westman, PhD (MRI Core, Karolinska Institutet), Lars-Olof Wahlund, MD PhD (MRI Core, Karolinska Institutet), Per Wollmer, MD PhD (PET Core, Lund University), (PET Core, Lund University), Ruben Smith, MD PhD (PET Core, Lund University), Tomas Olsson, PhD (PET Core, Lund University).
