# Supplementary material for: Blood-based NfL: A biomarker for differential diagnosis of parkinsonian disorder
Source: Neurology. 2017 Mar 7;88(10):930–7. doi: 10.1212/WNL.0000000000003680 (PMC5333515; doi:10.1212/WNL.0000000000003680)
Supplement: Accompanying Editorial [file supp_WNL.0000000000003680_922.pdf]

EDITORIAL

Neurofilament light

A heavyweight diagnostic biomarker in neurodegenerative parkinsonism?

Guido Alves, MD, PhD
Laura Bonanni, MD, PhD

Correspondence to Dr. Alves: algu@sus.no

Neurology® 2017;88:922–923

Neurodegenerative parkinsonian disorders were historically divided into 2 major groups based on their clinical picture, treatment response, and prognosis. One group encompassed Parkinson disease (PD), characterized at onset by usually asymmetrical extrapyramidal motor symptoms showing a marked and sustained response to levodopa, and subsequent slow motor decline over time. The other group included the much rarer atypical parkinsonian disorders (APDs), including multiple system atrophy (MSA), progressive supranuclear palsy (PSP), and corticobasal degeneration (CBD), with features signaling early autonomous, pyramidal, or cortical involvement, poor motor response to levodopa, and a much more rapid functional decline and higher mortality than usually seen in PD.[1]

This clinical black-and-white world has turned out to be much more shaded in light of major advances in our understanding of the neurobiology and genetics of movement disorders. Neurodegenerative parkinsonian diseases, even though characterized by heterogeneous pathophysiologic mechanisms (PD and MSA are synucleinopathies, PSP and CBD are tauopathies) and often a different genetic background,[2] share several clinical features, making it difficult to differentiate the single diseases. This is particularly true in early disease stages when the red flags suggestive of APDs sometimes are subtle or even absent. Hence, the clinical distinction of PD and APDs represents a challenge, and high misdiagnosis rates are often reported in the literature.[3,4]

Structural and functional neuroimaging can enhance diagnostic accuracy in parkinsonian disorders, but are costly and, in the case of SPECT and PET, usually available only at specialized centers. The research community has therefore been searching for biomarkers to aid in the differential diagnosis of parkinsonian disorders. CSF obtained by lumbar puncture has traditionally been the most promising biomarker source in neurodegenerative diseases. However, the availability of a blood-based diagnostic marker would clearly be an advantage, given that venipuncture is less invasive and can be easily performed even in primary centers.

Neurofilament light chain protein (NfL) is an important component of the neural cytoskeleton, and crucial for axon structure, transport, and growth.[5,6] Increased levels of NfL in CSF are considered a marker of axonal neuronal damage and are valuable in the discrimination of PD from APDs.[6] In addition, recent studies indicate that CSF NfL levels in PD increase with disease duration,[7] although they do not correlate with worsening of clinical features.[8] As a timely and important extension, Hansson et al.,[9] in this issue of *Neurology*®, take this research a step forward, investigating the potential of NfL as a blood-based biomarker in the differential diagnosis of these major diagnostic groups. Using a recently developed ultrasensitive single molecule array (Simoa) immunoassay, they found that blood NfL can distinguish between PD and APDs with high diagnostic accuracy. The results presented[9] seem convincing for several reasons. First, the study includes 3 independent cohorts from 2 different countries, comprising more than 500 individuals. Second, NfL levels in blood appear to be highly reliable, because they strongly correlate with those in CSF in a large subset of patients ($n > 300$). This may allow the use of blood NfL measurements in clinical trials, where repeated measures may be necessary to follow the rate of axonal degeneration, overcoming the ethical and procedural difficulties in performing serial lumbar punctures. Third, blood NfL concentrations discriminate between PD and APDs even at the early stage of disease, when decisions should be made about access to clinical trials, best treatment choice, and prediction of disease course. Overall, blood NfL seems to have the potential to provide a good biomarker to differentiate PD from APD, with potentially high usefulness in the clinical setting.

Some methodologic limitations and issues relevant to the clinical application of these findings are worth mentioning. NfL is intrinsically nonspecific in terms of the pathologic process underlying neurodegeneration. Indeed, increased CSF levels of NfL were first reported in amyotrophic lateral sclerosis[10] and subsequently across a range of various conditions, including Alzheimer disease, frontotemporal dementias, multiple sclerosis, cerebrovascular disease, traumatic

See page 930

From The Norwegian Centre for Movement Disorders (G.A.) and Department of Neurology (G.A.), Stavanger University Hospital, Norway; and the Department of Neuroscience Imaging and Clinical Sciences (L.B.), University G. d'Annunzio of Chieti-Pescara, Italy.

Go to Neurology.org for full disclosures. Funding information and disclosures deemed relevant by the author, if any, are provided at the end of the editorial.

© 2017 American Academy of Neurology

© 2017 American Academy of Neurology. Unauthorized reproduction of this article is prohibited.

brain injury, and neuropathic disorders.[5] Therefore, alterations of this marker in blood or CSF have to be put into clinical context. In addition, Hansson et al.[9] find increased NfL levels of similar magnitude in MSA, PSP, and CBD. Thus, NfL cannot separate these disorders, which would be desirable from a clinical point of view. Further, most patients with PD displayed NfL levels in the normal range, meaning that we still lack an easily accessible disease-specific diagnostic biomarker for the most common movement disorder. Finally, although Hansson et al.[9] carefully diagnosed patients on clinical grounds, the lack of pathologic confirmation in most cases has to be considered a (difficult-to-avoid) limitation in these clinically overlapping disorders. In addition, the authors used a research grade assay to determine NfL and did not provide cutoff values to distinguish between the diagnostic groups. Further steps needed towards clinical implementation therefore include the development of a clinical grade assay and the determination of cutoff values to define the presence of abnormal NfL levels (suggestive of APDs). Ideally, this should be done in patients whose diagnosis is pathologically proven, but also in those in the very earliest disease stages, including the premotor phase.

The study by Hansson et al.[9] represents an important step towards the development of a clinically useful and easily accessible blood-based biomarker for differentiating PD from APDs, parkinsonian disorders with similar clinical appearance but with different prognosis. Distinguishing these major parkinsonian groups is crucial for best possible treatment and care, and not least for providing adequate information to patients and caregivers on their future needs and perspectives. When disease-modifying treatments become available, diagnosing parkinsonian disorders correctly at early, possibly preclinical stages will be even more important.

STUDY FUNDING

No targeted funding reported.

DISCLOSURE

The authors report no disclosures relevant to the manuscript. Go to Neurology.org for full disclosure forms.

REFERENCES

1. Fielding S, Macleod AD, Counsell CE. Medium-term prognosis of an incident cohort of parkinsonian patients compared to controls. Parkinsonism Relat Disord 2016; 32:36–41.
2. Albin RL, Dauer WT. Parkinson syndrome: heterogeneity of etiology; heterogeneity of pathogenesis? Neurology 2012;79:202–203.
3. Caslake R, Moore JN, Gordon JC, Harris CE, Counsell C. Changes in diagnosis with follow-up in an incident cohort of patients with parkinsonism. J Neurol Neurosurg Psychiatry 2008;79:1202–1207.
4. Rizzo G, Copetti M, Arcuti S, Martino D, Fontana A, Logroscino G. Accuracy of clinical diagnosis of Parkinson disease: a systematic review and meta-analysis. Neurology 2016;86:566–576.
5. Yuan A, Rao MV, Veeranna, Nixon RA. Neurofilaments at a glance. J Cel Sci 2012;125:3257–3263.
6. Hall S, Ohrfelt A, Constantinescu R, et al. Accuracy of a panel of 5 cerebrospinal fluid biomarkers in the differential diagnosis of patients with dementia and/or parkinsonian disorders. Arch Neurol 2012;69:1445–1452.
7. Hall S, Surova Y, Ohrfelt A, et al. Longitudinal measurements of cerebrospinal fluid biomarkers in Parkinson's disease. Mov Disord 2016;31:898–905.
8. Hall S, Surova Y, Ohrfelt A, Zetterberg H, Lindqvist D, Hansson O. CSF biomarkers and clinical progression of Parkinson disease. Neurology 2015;84:57–63.
9. Hansson O, Janelidze S, Hall S, et al. Blood-based NfL: a biomarker for differential diagnosis of parkinsonian disorder. Neurology 2017;88:930–937.
10. Rosengren LE, Karlsson JE, Karlsson JO, Persson LI, Wikkelso C. Patients with amyotrophic lateral sclerosis and other neurodegenerative diseases have increased levels of neurofilament protein in CSF. J Neurochem 1996; 67:2013–2018.

© 2017 American Academy of Neurology. Unauthorized reproduction of this article is prohibited.
